# Supplementary material for: MetaRibo-Seq measures translation in microbiomes
Source: Nat Commun. 2020 Jun 29;11:3268. doi: 10.1038/s41467-020-17081-z (PMC7324362; doi:10.1038/s41467-020-17081-z)
Supplement: Supplementary file 10 — Supplementary Data 7 [file 41467_2020_17081_MOESM10_ESM.zip › File2/Confidence_High_Taxonomy/357032_out.krona.html]

Javascript must be enabled to view this page.

members
magnitude
magnitudeUnassigned
count
unassigned
taxon
rank

357032\_out

15

2
15
superkingdom

15
1239
phylum

2
class
186801

2
186802
order

2
family
216572

2
459786
genus

species
2

SRS042628\_contig\_number\_7031SRS055017\_contig\_number\_15285
1945593

13

SRS011134\_contig\_number\_contig-100\_1927.284203SRS016018\_contig\_number\_contig-100\_4977.4977SRS019068\_contig\_number\_104664SRS050998\_contig\_number\_contig-100\_10920.10921SRS052697\_contig\_number\_24647SRS077024\_contig\_number\_contig-100\_2680.92781SRS077392\_contig\_number\_9277SRS1041033\_contig\_number\_contig-100\_1428.152239SRS1041141\_contig\_number\_contig-100\_14023.14024SRS142503\_contig\_number\_contig-100\_7246.205576SRS144362\_contig\_number\_38392SRS146812\_contig\_number\_22161SRS149784\_contig\_number\_20976
1897031
species
